# Accumulation and Connectivity of Coarse Woody Debris in Partial Harvest and Unmanaged Relict Forests

**DOI:** 10.1371/journal.pone.0113323

**Published:** 2014-11-19

**Authors:** Robert C. Morrissey, Michael A. Jenkins, Michael R. Saunders

**Affiliations:** 1 Department of Forest Ecology, Faculty of Forestry and Wood Sciences, Czech University of Life Sciences, Prague, Czech Republic; 2 Hardwood Tree Improvement and Regeneration Center, Department of Forestry and Natural Resources, Purdue University, West Lafayette, Indiana, United States of America; Ecole Pratique des Hautes Etudes, France

## Abstract

When a tree dies, it continues to play an important ecological role within forests. Coarse woody debris (CWD), including standing deadwood (SDW) and downed deadwood (DDW), is an important functional component of forest ecosystems, particularly for many dispersal-limited saproxylic taxa and for metapopulation dynamics across landscapes. Processes, such as natural disturbance or management, modify forest composition and structure, thereby influencing CWD abundance and distribution. Many studies have compared older forests to forests managed with even-aged silvicultural systems and observed a prolonged period of low CWD occurrence after harvesting. With fine-scale spatial data, our study compares the long-term impacts of light partial harvesting on the CWD structure of eastern deciduous hardwood forests. We mapped and inventoried DDW and SDW using variable radius plots based on a 10 m×10 m grid throughout an unmanaged, structurally-complex relict forest and two nearby forests that were partially harvested over 46 years ago. The relict stand had significantly larger individual pieces and higher accumulations of DDW and SDW than both of the partially harvested stands. Connectivity of CWD was much higher in the relict stand, which had fewer, larger patches. Larger pieces and higher proportion of decay-resistant species (e.g. *Quercus* spp.) in the relict forest resulted in slower decomposition, greater accumulation and increased connectivity of CWD. Partial harvests, such that occur with selection forestry, are generally considered less disruptive of ecosystem services, but this study highlights the long-term impacts of even light partial harvests on CWD stocks and distribution. When planning harvesting events, forest managers should also consider alternative methods to ensure the sustainability of deadwood resources and function.

## Introduction

Even after death, trees continue to influence ecological processes and biodiversity of forest ecosystems [1]. Coarse woody debris (CWD), both standing dead trees (SDW) and downed deadwood (DDW), provides regeneration substrate for some plants, serves as habitat for many insect and wildlife taxa, mitigates runoff and erosion from slopes, contributes to soil development, and provides long-term sequestration of carbon and other nutrients [2]. Because CWD decays slowly, it can potentially influence these ecological processes at local, stand, and landscape levels for many decades. As forest managers have attempted to integrate a broader array of ecological objectives into their planning, provision of CWD to preserve structural attributes and ecological processes has become increasingly emphasized.

The importance of CWD as habitat has been well documented for many organisms including birds [3], arthropods [4], fungi [5], and small mammals and amphibians [6]. Given the abundance of CWD in mature forests, it is conceivable that many species evolved to rely on a suite of habitats based on abundance, species, size, decay stages, and position (standing vs. fallen) of CWD [1], [2]. In these forests, CWD-dependent species with limited dispersal should predominate, and the connectivity and spatial arrangement of CWD should be critical to the abundance of species and individuals [7]. Availability, connectivity, patch size and abundance of CWD are critical to supporting and maintaining diverse natural communities [8]. Connectivity, an emergent property across multiple ecological scales, strongly influences the population and metapopulation dynamics of multiple taxa.

Not surprisingly, the literature is replete with differences among species regarding the importance of connectivity of CWD at the stand scale [5], [7], [9]. Organisms that are highly mobile are likely unaffected at stand-level scales [10], but there may be population fitness benefits associated with high connectivity for these species, e.g. prey avoidance, perch sites [11]. For saproxylic taxa that may be dispersal-limited, including many insects, bryophytes, lichens, amphibians, and reptiles, the scale of connectivity or, conversely, fragmentation, may influence an individual’s ability to disperse and occupy new habitat [12], [13], [14]. Thus, changes to spatiotemporal patterns of habitat continuity in managed and fragmented forests likely influence species dispersal, success, and fitness.

Understanding the spatiotemporal dynamics of CWD is essential to framing strategies for forest and wildlife management [15], and is thus an essential element of biological conservation in forest ecosystems. Spatiotemporal CWD dynamics are largely influenced by tree mortality and decay rates. Tree mortality is not evenly distributed over time or space [1] and is related to disturbance history, stand structure, and site factors [2], all of which influence patterns of CWD dispersion. Several studies of old-growth forests have observed high abundance and aggregated patterns of DDW [5], [7], [10], but patterns of SDW varied across many studies [16], [17]. Light partial harvests, which include thinnings, selection cut, and some timber stand improvement treatments, generally disproportionally remove low vigor and individual or small groups of trees, in order to favor more vigorous, higher quality trees throughout the stand. Therefore, these harvests may impact future spatiotemporal inputs of CWD by influencing CWD size, species, volume, input rate, and spatial distribution, which thereby can both reduce the abundance of CWD over time and either homogenize or concentrate CWD distribution, depending on the management regime. Light partial harvests have typically been considered to have a short-term and minor ecological impact, but studies of CWD impacts related to known management events is critical to understand long-term dynamics of ecological processes.

We examined the quantity, quality, and spatial arrangement of deadwood in a relict forest and two managed forests that were partially harvested more than 46 years ago. All stands are isolated forests within a fragmented agricultural matrix; thus, connectivity at a stand level may be especially important to sustain dispersal-limited taxa under the assumption that highly connected populations are less likely to succumb to local random extinction events. We chose not to consider individual species or taxa in this study, but rather focus on structural connectivity of the CWD within stands. Based on differences in disturbance histories of these stands, we hypothesized: 1) the overall volume of CWD would be less in managed stands; 2) average piece size and abundance of CWD would be less in managed stands than in the relict, unmanaged stand; 3) the distribution of CWD volume among decay stages would shift towards less decayed material in managed stands relative to the relict stand; and 4) distribution of CWD would exhibit higher levels of connectivity in less disturbed stands.

## Methods

### Study sites

Study sites were located on the Davis Purdue Agricultural Center property; permissions were granted by the Woodlands Management Committee through the Department of Forestry and Natural Resources of Purdue University. They included three *Quercus*-dominated deciduous forest stands within a fragmented, agricultural landscape in east-central Indiana, U.S.A. (40°15′26″, 85°09′16″). These stands have gently rolling, low relief topography with elevation changes <5 m across the entire area. Since the middle of the 20^th^ century, all stands have been impacted by Dutch elm disease and elm phloem necrosis, both of which resulted in the death of most elm trees before they recruit into the overstory canopy. The relict forest is the largest stand (20.6 ha), of which we sampled the 8.5 ha interior. Records indicate that this stand has not been managed, except for the removal of some dead and dying trees in 1941, 1948, and 1955 (volume removed of 5.3 m^3^ ha^−1^). The smallest stand (2.8 ha) was sampled in its entirety; this stand, referred to as the double-harvest stand henceforth, was selectively harvested in 1951 (volume removed of 9.8 m^3^ ha^−1^) and again in 1964 (volume removed of 7.8 m^3^ ha^−1^). The third stand (2.9 ha) was also selectively harvested in 1951 (volume removed of 8.1 m^3^ ha^−1^); this stand will henceforth be referred to as the single-harvest stand. According to historical records, all trees removed from these stands were overstory individuals ≥40 cm at 1.37 m (DBH). For a more thorough description of these sites, see [18].

### Data collection

We sampled CWD on a 10 m by 10 m grid using probability proportional to size (PPS) sampling methods. At each grid point, DDW was sampled using point relascope sampling [19] with an angle of 60°, and SDW was sampled using a 10-factor prism**.** For each individual DDW piece with a small end diameter (SED) ≥10 cm diameter, we measured the large end diameter (LED), SED, and the length of the piece. When pieces were long enough to still have intact multiple branches within a partially intact crown, we chose the single most linear piece to measure the SED and length. In rare cases, branches of intact crowns had sufficiently large diameters and were sampled as independent pieces. For SDW, pieces had to be ≥1.3 m in height, but no minimum diameter limit was used; height was recorded to either top of crown, if still intact, or top of the standing stem.

To estimate individual DDW piece volumes, we assumed a conic-paraboloid shape to minimize bias of estimates and field measures required [20]. We calculated volume (V; m^3^) using:
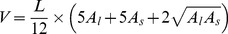
(5.1)where L is DDW length (m), A_l_ is cross-sectional area (cm) at large end, and A_s_ is cross-sectional area (cm) at small end. Because we were only interested in historical inputs of DDW as a function of forest dynamics, we did not account for decreased volumes of collapsed pieces in advanced states of decay, as recommended by [20]. Volume for SDW with intact or partially intact crowns was based on unpublished local volume tables using a 10 cm top diameter (Table S1). For sheared stems, volume was estimated with equation 5.1 using height for length, breast height diameter to calculate A_l_, and a form class of 78 was used to estimate SED and calculate A_s_; form class mathematically describes the shape of the tree bole and provides an estimate of the bole diameter at any given length (or height in this case) based on the taper of the bole and DBH [21].

We classified each piece according to its decay stage; when a piece is sufficiently large, it often had different sections of the piece in differing decay stages, so we assigned decay stage based on the section most representative of the piece based on volume. For DDW, we classified decay stage as: ‘sound’ if recently dead with wood still hard and bark largely present, either loose or tight; ‘intermediate-decay’ as bark mostly gone, bole periphery softened whereby a blade can penetrate the outer layer; and ‘high-decay’ as little to no bark remains, bole periphery is very soft and extends in to the core, often partially incorporated into forest floor or vegetation has begun to colonize. For SDW, we had only two categories: ‘sound’ and ‘intermediate-decay’, as described above.

### Data analyses

We compared mean length, LED, and volume of individual DDW among all three stands using a generalized linear model (GLM) to account for the unbalanced number of pieces sampled in each stand. We used a log transformation of the response variable and a Gaussian distribution of errors. The same procedure was used for individual SDW pieces to examine DBH, height, and volume. To test for differences of DDW and SDW abundance among stands, we used a subset of the sampling grid (30 m by 30 m) distribution to limit correlation among plots related to sampling of individual pieces from multiple different plots. We used a GLM procedure on the square root transformed response variable and a Gaussian distribution of errors. Post-hoc tests of comparison were conducted using Tukey’s honest significant differences method.

We tested for differences between stands based on volume ha^−1^ by size classes (≤30 cm, >30 cm and ≤60 cm, >60 cm) and decay stages using a chi-square test. For density, we could only test for differences in the number of pieces of DDW by decay stages; DDW by size classes and SDW for decay and size classes had small bin values that could not be tested formally, although we do present them graphically.

Estimates of connectivity were developed to characterize the potential for dispersal-limited organisms to traverse the stand based on their affinity for CWD of a certain form or size. We assumed organisms could disperse 10 m, or the distance between two grid cells, if CWD was present in adjacent cells; thus, data included SDW and DDW pieces that were tallied from at least two gridpoints. Connectivity was defined by the number of gridpoints that tallied a CWD piece divided by the total number of gridpoints, and reported as a percentage by diameter size classes (10 cm width). We standardized patch area and patch density using per hectare values; patches of CWD were defined either by the edge of the sample area, adjacent plots that tallied no CWD, or some combination of the two. Connectivity interactions between DDW and SDW were not substantiated because the PPS methods differ both in angle used to select sample pieces (i.e., DDW is sampled with 60° and SDW is sampled with 1° 48′) and also the size criterion used for each sample procedure (i.e., DDW uses length of piece and SDW uses diameter).

Given the time since harvest in these stands and the lack of stumps, we assumed that all CWD was related to natural processes rather than the harvest events. All tests were conducted with an alpha level of 0.05, and all analyses were done using R [22].

## Results

Individual pieces of DDW in the relict stand were significantly larger in LED, length, and volume compared to the partial-harvest stands (Table 1; all p≤0.003). Mean volume of individual DDW pieces within the relict stand was 142% larger compared to the double-harvest stand and 248% larger than those of the single-harvest stand. Individual SDW pieces of the relict and single-harvest stands were not significantly different (all p≥0.202); however, individual piece means of DBH, height, and volume of both stands differed from those of the double-harvest stand (all p≤0.02). Within the double-harvest stand, the population of SDW was dominated by smaller pieces with correspondingly low volumes.

**Table 1:** Coarse woody debris piece summaries.

a) Downed deadwood				
Tract	n	LED (cm)	Length (m)	Volume (m^3^)
Relict	924	34.3±0.7 A	11.2±0.2 A	1.19±0.08 A
Single-harvest	288	26.0±0.9 B	8.7±0.2 B	0.48±0.06 B
Double-harvest	153	31.3±2.2 B	7.6±0.3 C	0.84±0.21 B
**b) Standing deadwood**				
**Tract**	**n**	**DBH (cm)**	**Height (m)**	**Volume (m^3^)**
Relict	182	46.8±2.5 A	13.2±0.7 A	2.27±0.26 A
Single-harvest	96	36.5±2.4 A	10.9±0.8 A	1.24±0.28 A
Double-harvest	57	28.7±3.7 B	7.0±0.5 B	0.56±0.16 B

Total number of pieces (n) and mean (± standard error) of individual piece sizes of sampled a) downed deadwood (minimum diameter of 10 cm) and b) standing deadwood in relict, single-harvest, and double-harvest stands. Large end diameter (LED), length, and volume of downed deadwood, and diameter at 1.37 m (DBH), total height, and volume of standing deadwood are presented. We tested for differences (p<0.05) of log-transformed values using a generalized linear model and post-hoc multiple comparisons with Tukey’s honestly significant difference. Different letters indicate significant differences among stands.

Mean number of DDW pieces ha^−1^ within the relict stand was significantly higher compared to the single-harvest stand (p = 0.033), but did not differ from the double-harvest stand (p = 0.134; Table 2); the partial-harvest stands were not significantly different from one another (p = 0.975). Significant differences between the relict and partial-harvest stands were evident for volume ha^−1^ of DDW (all p<0.001). The relict stand had 328% and 219% more volume compared to the single-harvest and double-harvest stands, respectively; the partial-harvest stands were not significantly different from one another (p = 0.918). Mean density of SDW pieces ha^−1^ did not differ among all three stands (all p≥0.764), but SDW volume ha^−1^ of the relict stand was significantly higher (both p≤0.008) than that of both partial-harvest stands, which were not different from one another (p = 0.773). Although density of SDW pieces ha^−1^ did not significantly differ among stands (all p<0.245), the double-harvest stand had 41.2±9.1 (mean ± SD) pieces ha^−1^ on average, almost double the relict stand. The SDW volume ha^−1^ of the relict stand was 217% greater than that of the single-harvest stand and 320% greater than that of the double-harvest stand.

**Table 2:** Coarse woody debris stand-level summaries.

a) Downed deadwood			
Tract	n	Pieces ha^−1^	Volume ha^−1^ (m^3^ ha^−1^)
Relict	817	242.8±17.0 A	130.8±4.2 A
Single-harvest	392	154.8±14.8 B	39.9±2.5 B
Double-harvest	234	199.3±28.1 AB	59.8±6.4 B
**b) Standing deadwood**			
**Tract**	**n**	**Pieces ha^−1^**	**Volume ha^−1^ (m^3^ ha^−1^)**
Relict	817	21.7±1.9 A	24.3±1.0 A
Single-harvest	392	18.4±2.1 A	11.2±0.7 B
Double-harvest	234	41.2±9.1 A	7.6±0.7 B

Total plots (n), and mean (± standard error) number of pieces ha^−1^ and volume ha^−1^ of a) downed deadwood (minimum diameter of 10 cm) and standing deadwood in relict, single-harvest, and double-harvest stands. To minimize correlation between plots, we used a subset of plots based on 30 m×30 m spacing in each stand. We tested for differences (p<0.05) of square root-transformed values using a generalized linear model and post-hoc multiple comparisons with Tukey’s honestly significant difference. Different letters indicate significant differences among stands.

**Figure 1:**
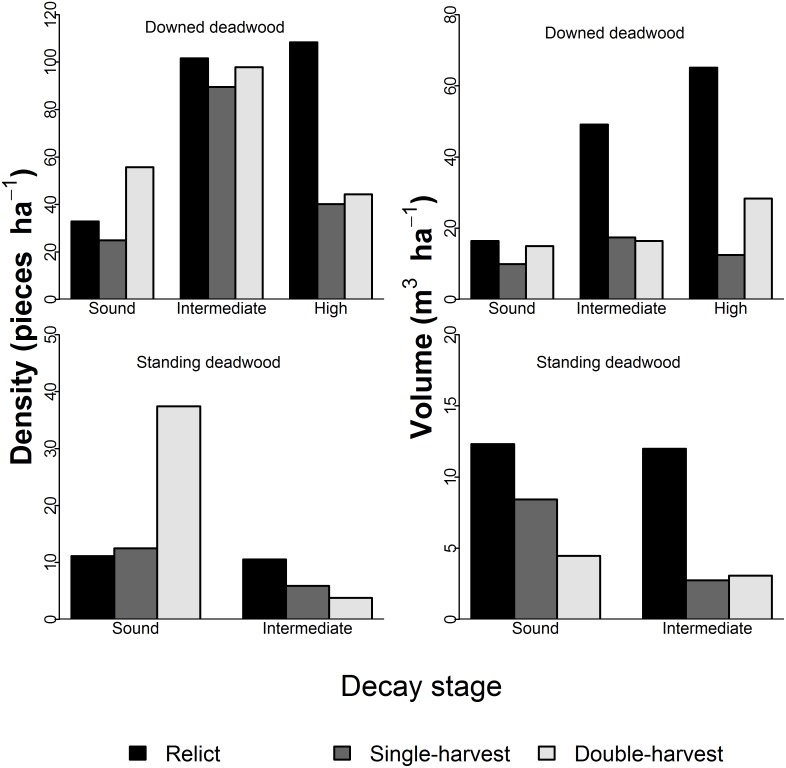
Coarse woody debris decay stage summaries. Downed deadwood (DDW; top row) and standing deadwood (SDW; bottom row) number of pieces ha^−1^ and volume ha^−1^ by decay stage for relict, single-harvest, and double-harvests stands. Decay stages were assigned as: sound, intermediate, or high; for SDW, sound and intermediate categories were used. For a more thorough description, see 2.2 Data Collection.

We observed a significant difference in the number of DDW pieces ha^−1^ (χ^2^ = 15.2, p = 0.004) and volume ha^−1^ (χ^2^ = 131.0, p<0.001) by decay stages among stands (Figure 1). In the relict stand, density of high-decay DDW pieces in the relict stand (108.4 pieces ha^−1^) was much higher than either of the partial-harvest stands (≤44.4 pieces ha^−1^), and volumes in intermediate- and high-decay, 49.4 m^3^ ha^−1^ and 65.1 m^3^ ha^−1^, respectively, were more than twice the amount in either of the partial-harvest stands. Density of SDW pieces by decay stage could not be tested because there were too few pieces in the intermediate-decay stage, but there was more than three times the number of sound SDW pieces (37.4 pieces ha^−1^) in the double-harvest stand compared to the other stands (Figure 1). Volume ha^−1^ of SDW by decay stage was significantly different among stands (χ^2^ = 177.6, p<0.001); the relict stand had 12 m^3^ ha^−1^ in the intermediate decay stage compared to <5 m^3^ ha^−1^ in each of the partial-harvest stands.

**Figure 2:**
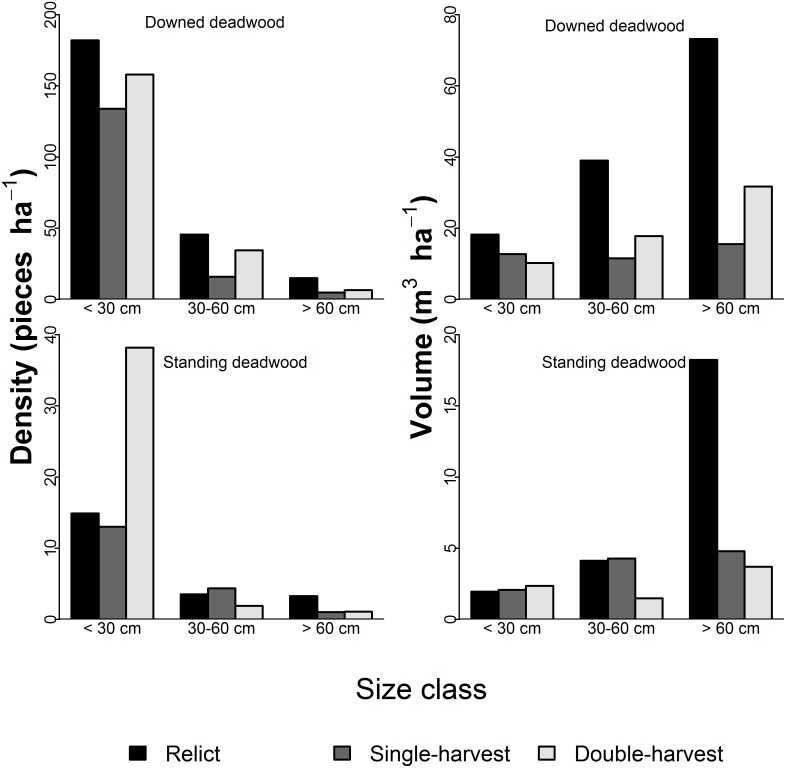
Coarse woody debris size class summaries. Downed deadwood (DDW; top row) and standing deadwood (SDW; bottom row) number of pieces ha^−1^ and volume ha^−1^ by size class for relict, single-harvest, and double-harvests stands. Size classes were defined as diameters of ≤30 cm, >30 cm and ≤60 cm, and >60 cm; DDW diameter was based on large end diameter, and we used diameter at 1.37 m (DBH) for SDW.

For size class distributions, we could not test for differences in the number of DDW or SDW pieces ha^−1^ among stands because the largest size classes had very low values (Figure 2). All stands exhibited high densities of DDW <30 cm, similar to the living tree distribution [18]. The double-harvest stand had more than double the density of SDW pieces, 38.2 pieces ha^−1^, in the <30 cm size class compared to the other two stands. Volume of DDW ha^−1^ across size classes was different across stands (χ^2^ = 103.8, p<0.001), with the relict stand having larger volumes in all size classes, most notably in classes >30 cm. Volume of SDW ha^−1^ across size classes differed across stands (χ^2^ = 76.3, p<0.001), attributable to the large volume of large diameter (≥60 cm) SDW, 18.2 m^3^ ha^−1^, compared to 4.8 m^3^ ha^−1^ and 3.7 m^3^ ha^−1^ for the single- and double-harvest stands, respectively.

**Figure 3:**
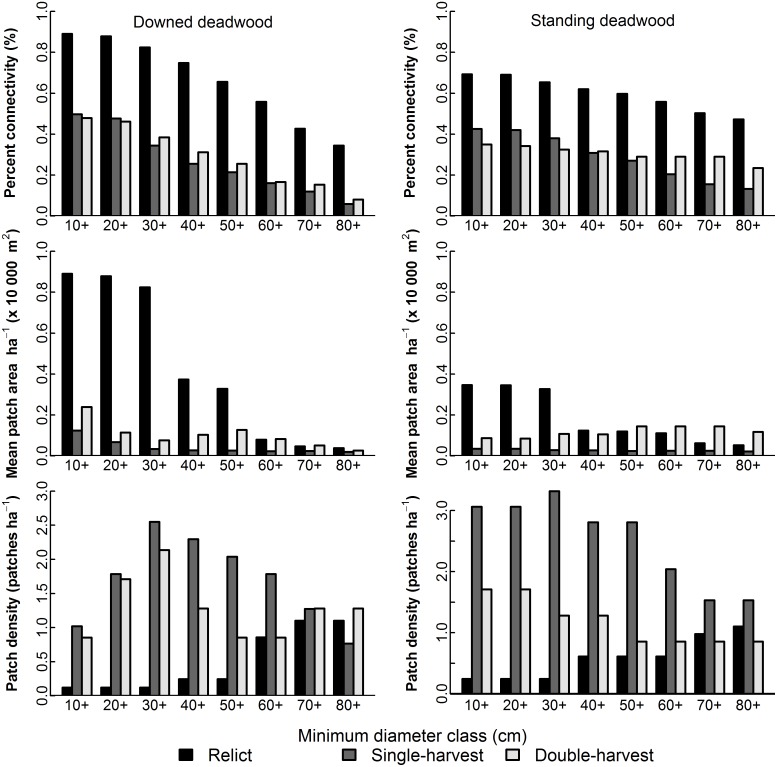
Coarse woody debris connectivity and patch-level summaries. Percent of all plots connected based upon presence of CWD (top row), mean patch area ha^−1^ (middle row), and number of patches ha^−1^ (bottom row) by 10 cm diameter classes for relict, single-harvest, and double-harvests stands. Downed deadwood (left column) diameter was based on large end diameter, and we used diameter at 1.37 m (DBH) for standing deadwood (right column); each diameter class indicates pieces of indicated size and larger were included in the analysis.

**Figure 4:**
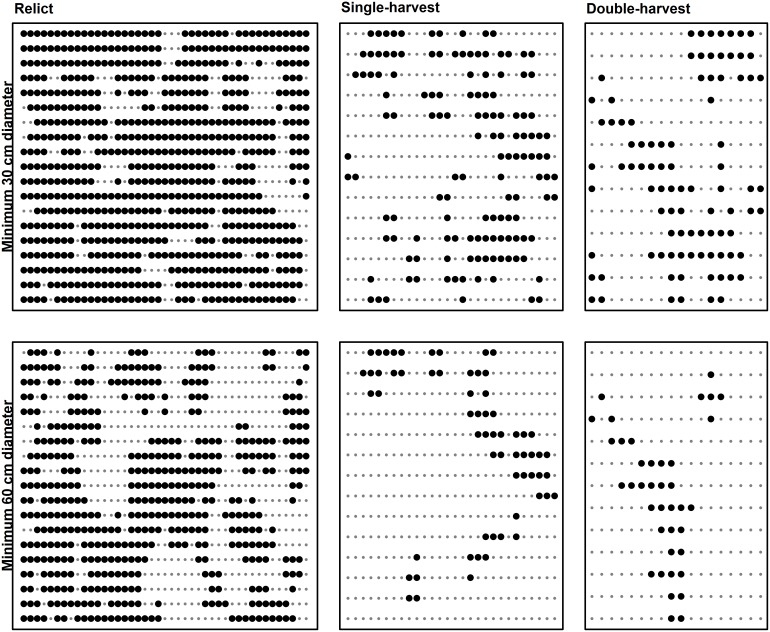
Downed deadwood connectivity examples. Display of connected plots for downed deadwood (DDW) in relict, single-harvest, and double-harvests stands for pieces ≥30 cm (top row) and ≥60 cm (bottom row) based on large end diameter. Large dots indicate plots that sampled at least one DDW piece of the stated size, and small dots indicate plots with no DDW pieces of designated size sampled.

**Figure 5:**
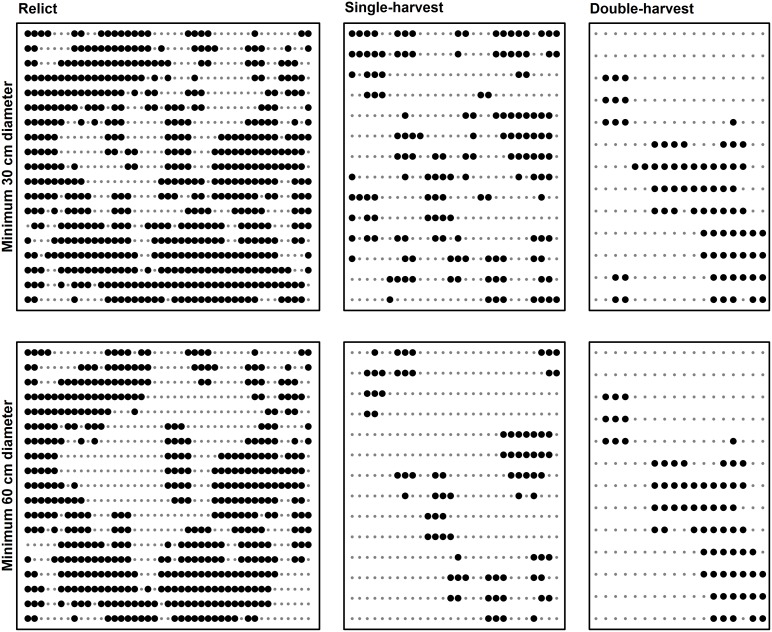
Standing deadwood connectivity examples. Display of connected plots for standing deadwood (SDW) in relict, single-harvest, and double-harvests stands for pieces ≥30 cm (top row) and ≥60 cm (bottom row) based on diameter at 1.37 m height. Large dots indicate plots that sampled at least one SDW piece of the stated size, and small dots indicate plots with no SDW pieces of designated size sampled.

**Figure 6:**
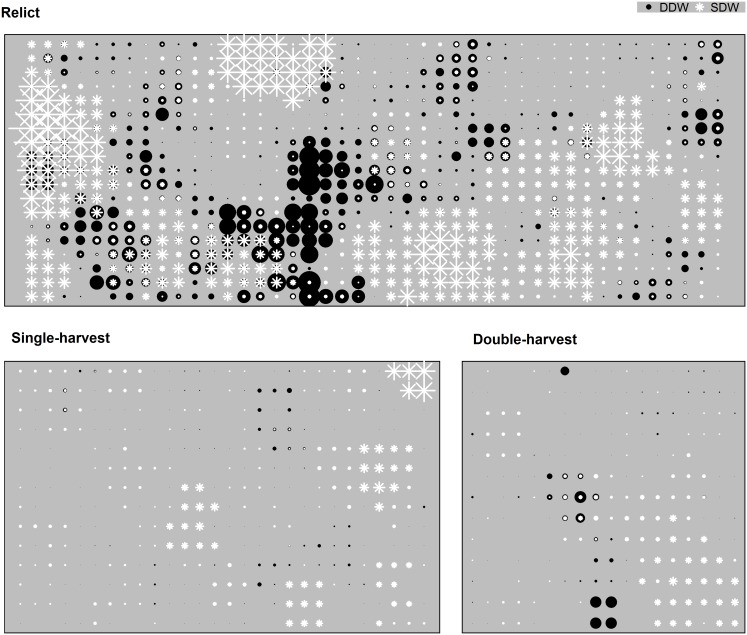
Coarse woody debris spatial distributions by volume. Each panel displays the volume (m^3^) of downed deadwood (DDW; black circles) and standing deadwood (SDW; white stars) sampled at each plot location for relict, single-harvest, and double-harvests stands; size of symbol within each pane is standardized.

Connectivity of DDW and SDW declined as minimum diameter increased in a similar pattern for all stands, but the relict stand always displayed much higher connectivity (Figures 3, 4, and 5). Mean patch area ha^−1^ of DDW generally decreased as minimum diameter increased, but the partial-harvest stands area did not vary as much by minimum diameter class compared to the relict stand (Figure 3). Mean patch area ha^−1^ of the relict stand DDW exhibited two abrupt decreases at minimum LEDs of 40 cm and 60 cm; beyond 60 cm, all three stands had similar mean patch area ha^−1^. For SDW, patch sizes show a similar flat profile for partial-harvest stands, and mean patch area ha^−1^ of the relict stand abruptly decreases beyond a diameter of 40 cm. Number of patches ha^−1^ generally shows an inverse relationship to mean patch area ha^−1^, and the partial-harvest stands tended to have a greater number of smaller patches (Figures 4 and 5). Beyond a diameter of 60 cm for DDW and SDW, number of patches was more similar across all stands, although the single-harvest stand generally exhibited a greater number of patches than the other two stands. Although we used no metrics to compare overlay of DDW and SDW sample volume plot^−1^, differences between stands were apparent (Figure 6). In the relict stand, plots with large SDW volumes tend to occur in areas with little or no DDW; we observed a similar pattern in the partial-harvest stands, but volumes of DDW and SDW throughout the stands were much lower. There were very few plots with no DDW or SDW sampled in the relict stand, but there are numerous patches throughout the partial-harvest stands with little or no DDW or SDW sampled.

## Discussion

Almost half a century later, the effects of light partial harvesting on the type, amount, and spatial distribution of CWD remains evident. The relict forest had significantly larger pieces, greater volume, and higher connectivity of DDW and SDW compared to both partially harvested stands. Partial harvesting has altered species composition and structure relative to the relict stand [18], and the abundance and spatial distribution of CWD are a function of the resulting mortality and decomposition rates [23], [24].

Density-independent mortality related to windthrow, senescence, disease, insects, or some combination of these agents occurred in all stands, but they may affect species and/or different size trees in differing capacities [25]. For example, Dutch elm disease has been present in all stands since about the 1950s [26]. *Ulmus* trees, most of which were <20 cm DBH, were more abundant in the double-harvest stand (86 trees ha^−1^), but were also common in the relict (41 trees ha^−1^) and single-harvest stands (25 trees ha^−1^). Given the relatively small size of *Ulmus* trees in the understory, they are a relatively ephemeral source of CWD throughout all stands with limited impact on long-term volumes and connectivity. In contrast, windthrow and senescence were likely the primary disturbance types in the relict stand given the higher density of larger trees compared to the partially harvested stands [18]. Relict stand CWD volume ha^−1^ was significantly higher than both partially harvested stands similar to several other studies [27], [28], [29]. This observation is presumably related to the changes in the size distribution of living trees, and the associated change in potential exogenous natural disturbances, particularly wind, related to tree size.

Removal of individual or small groups of large canopy trees allows for the release, growth and development of advance regeneration, or the establishment of new regeneration. These areas tend to have higher stem densities of smaller stems that will experience endogenous disturbances, particularly density-dependent mortality, as the stand develops rather than exogenous natural disturbances. Although these areas may contribute numerous CWD pieces, their cumulative low volume dictates a more ephemeral source of CWD because of faster rates of decomposition [2], [29]. Using a chronosequence approach, one study [30] indicated that CWD dynamics of managed stands were more unidirectional related to succession in contrast to virgin forests that exhibited a more cyclical pattern. In boreal mesic forests of southern Finland, researchers observed larger average diameter pieces of DDW and SDW in old-growth stands versus managed stands, with considerably more large-diameter (≥40 cm) SDW in old-growth stands and twice as many CWD stems <10 cm in the managed stands compared to old-growth stands [27]. In boreal *Picea* forests of Sweden, significant differences in the availability of large diameter, highly-decayed CWD logs between selectively managed and semi-natural forests were observed, even after about 100 years [31]. In contrast, no differences were evident in DDW piece sizes in old-growth and selection cut stands of *A. saccharum*-dominated stands in Quebec, Canada, although SDW average diameter was lower in diameter-limit cuts [32]. Intermediate harvests can also influence species composition.

The overstory composition of the relict and partially harvested stands differed, which likely influenced decay rates and stand-level abundance of CWD. *Quercus* species dominated the overstory of all stands, but the relict stand *Quercus* basal area was 17.1 m^2^ ha^−1^ compared to only 7.9 m^2^ ha^−1^ and 8.6 m^2^ ha^−1^ in the single-harvest and double-harvest stands, respectively [18]. The partially harvested stands had a higher abundance of *Acer*, *Carya*, and *Fraxinus* individuals, which replaced the harvested overstory *Quercus* trees. At an old-growth deciduous forest in Indiana, U.S.A., it was predicted that 95% loss of CWD density of *Quercus*, *Carya*, and *Acer* species would occur at 171, 86, and 66 years, respectively [29]. Although we were unable to consistently identify CWD pieces to genus, the higher volumes of CWD within intermediate and high-decay stages likely reflects of the interaction of piece size and species composition within the relict stand; larger, more decay-resistant pieces will remain in situ compared to smaller pieces that decay quicker.

Rates of inputs (mortality events) and outputs (decomposition) are modified by management decisions, thus, CWD stores within a stand will also be modified. Assuming random mortality events with a 1% coefficient of variation over time [33], combined with slow decomposition rates related to large piece sizes and species composition, CWD can accumulate over time. This phenomenon may explain the aggregated patterns of CWD observed in one study [34] despite randomly dispersed mortality, as well as aggregated patterns of CWD reported for other old-growth forests [7], [10], [35]. Harvesting often alters rates of inputs and outputs, thus influencing potential CWD stores. One study observed that accumulation of CWD was slower after selective harvests, and that CWD levels had not returned to primeval forest levels even after 100 years [28]. Similarly, it has been proposed that selective logging operations interrupted the continuity of CWD inputs over time, thus influencing proportions of decaying logs and large CWD pieces [31]. Because there are fewer slow-decaying large pieces and a greater number of smaller CWD pieces derived from aggregated mortality events due to density-dependent competition within newly-formed harvest gaps, CWD is more ephemeral in selection harvests due to faster rates of decomposition of smaller pieces. Faster decomposition of CWD stores will result in lower volumes and decreased connectivity.

Decreased connectivity of DDW and SDW resulted in a larger number of smaller patches in the partially harvested stands, notably in diameter classes <60 cm. As minimum diameter class size increases, differences between all stands become minimized around 60 cm. Although this is partly an artifact of the methodology used whereby larger pieces are sampled more frequently across larger areas, connectivity, patch size, and number of patches were regulated by large diameter CWD. For taxa that can utilize smaller pieces, connectivity was still maintained in the in the partially harvested stands, being more than half of that observed in the relict stand, although the patch size and density were not comparable. Abundance of DDW and SDW sampled at each plot (Figure 6) indicated an inverse spatial relationship between the two pools of CWD; areas with abundant DDW tended to have low volumes of SDW and vice-versa. This may suggest a dynamic relationship between the two pools whereby SDW eventually contribute to DDW pools and enhances connectivity. In the partially harvested stands, large patches with little or no CWD are indicative of areas that were previously harvested but now contain vigorous trees; many of these areas will be devoid of large CWD for the foreseeable future.

Traditional forest management practices can reduce CWD stores in forest stands for extended periods of time; differences in CWD connectivity and abundance among the three stands remained evident 46+ years after harvesting. Because the partially harvested stands have received no management since the initial harvest, this scenario differs from conventional uneven-aged, selection management in the region where most trees larger than 60 cm are harvested, stands are entered at short intervals, often 10–15 years, and removals tend to include declining individuals. This management approach reduces CWD inputs and drives reduced accumulation and connectivity across the stand. Extended rotation and entry times are often cited as ecologically viable harvest practices that promote greater heterogeneity in forest structure. However, these options may not be economically feasible or sufficient to preserve CWD pools in many management regimes. Several other studies have observed low levels of CWD long after active management operations ceased [28], [30], [31], and it may take a century or more for CWD abundance to return to the range of natural levels without adequate planning. Retaining individual and groups of trees within harvested stands can help preserve current and future CWD associated dispersal-limited taxa, and enhance biodiversity [36]. Using an “island” approach (*sensu* [37]), within stands, especially in fragmented forest landscapes, may be more appropriate to preserve dispersal-limited, smaller populations and metapopulation dynamics across the landscape akin to the “lifeboat hypothesis”. We propose that differences in abundance and connectivity are attributable to decreased CWD stores in the managed stands. Although most studies of CWD focus on abundance within forests, understanding of CWD spatiotemporal dynamics may prove more critical to understand sustainability of CWD and its associated functions within forests.

## Supporting Information

Table S1 **Local volume table.**
(CSV)

Table S2 **Downed deadwood (DDW) data.**
(CSV)

Table S3 **Standing deadwood (SDW) data.**
(CSV)
